# Sangiangols A and B, Two New Dolabellanes from an Indonesian Marine Soft Coral, *Anthelia* sp.

**DOI:** 10.3390/molecules25173803

**Published:** 2020-08-21

**Authors:** Novriyandi Hanif, Anggia Murni, Junichi Tanaka

**Affiliations:** 1 Department of Chemistry, Faculty of Mathematics and Natural Sciences, IPB University, Bogor 16680, Indonesia; 2 Tropical Biopharmaca Research Center, IPB University, Bogor 16128, Indonesia; anggia_murni@apps.ipb.ac.id; 3 Department of Chemistry, Biology and Marine Science, University of the Ryukyus, Nishihara, Okinawa 903-0213, Japan; jtanaka@sci.u-ryukyu.ac.jp

**Keywords:** soft coral, dolabellane, cytotoxicity, NMR, molecular modelling

## Abstract

A new, rare trinor-dolabellane diterpenoid, sangiangol A (**1**), and one new dolabellane diterpenoid, sangiangol B (**2**), together with known cembranes and dolabellanes (**3**–**8**), were isolated from the ethyl acetate layer of an extract of an Indonesian marine soft coral, *Anthelia* sp. Compounds **1**–**8** exhibited moderate cytotoxicity against an NBT-T2 cell line (0.5–10 µg/mL). The structures of the new compounds were determined by analyzing their spectra and a molecular modelling study. A possible biosynthetic pathway for sangiangols A (**1**) and B (**2**) is presented. Cytotoxicity requires two epoxide rings or a chlorine atom, as in **4** (stolonidiol) and **5** (clavinflol B).

## 1. Introduction

Soft corals produce numerous, structurally diverse, biologically active terpenoids [1]. More specifically, Indonesian alcyonaceans are rich sources of diterpenoids with a variety of molecular skeletons. From 1970–2017, eight diterpenoid skeletons (briarane, cladiellane, *seco*-cladiellane, cembrane, *nor*-cembrane, dolabellane, flexibilane, and xenicane) were discovered in 11 genera of Indonesian alcyonaceans [2]. Among them, cembrane and briarane skeletons comprise a majority of the known diterpenoids in soft corals globally [2,3]. To date, soft corals of the genus *Anthelia*, family Xeniidae, have been shown to contain one type of sesquiterpenoid [4], three types of diterpenoids (xenicane [5], dolabellane [6], a C_24_-acetoacetylated diterpenoid [7,8]), and one type of steroid [9] with cytotoxic activity against various cell lines [4,5,6,7,8,9]. Moreover, the dolabellane stolonidiol (**4**), was identified as a promising candidate against Alzheimer’s disease after its mode of action in HEK293 cells was determined [10]. In our continuing study of metabolites of Indonesian *Anthelia* [4,6], we isolated known diterpenoids **3**–**8** and new dolabellanes, named sangiangols A (**1**) and B (**2**), the structures of which are the subject of this article.

## 2. Results and Discussion

**Figure 1:**
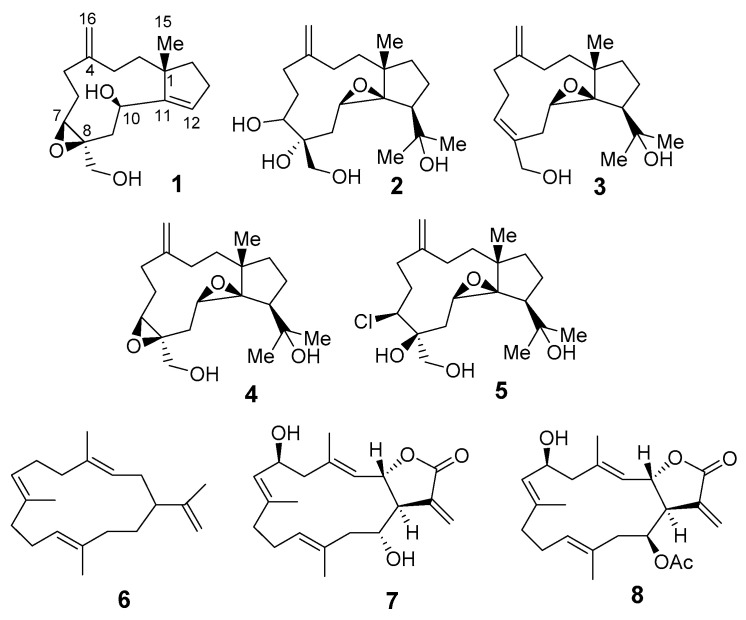
Chemical structures of dolabellane-type molecules (**1**–**5**) and cembrane-type molecules (**6**–**8**).

A sample of the soft coral *Anthelia* sp., collected at Banten (BTN) in northwestern Java, was thoroughly extracted with acetone. After concentration, the residue was partitioned between EtOAc and H_2_O. The EtOAc extract showed significant cytotoxicity against NBT-T2 cells at 1 μg/mL. Thus, it was chromatographed on silica gel, followed by normal or reversed phase HPLC to afford two new molecules, **1** and **2**, along with known compounds **3**–**8** (Figure 1).

Sangiangol A (**1**) was obtained as an optically active oil, [α]_D_^27^ + 20. Its molecular formula is C_17_H_26_O_3_ by HRESIMS and NMR (Table 1), indicating five degrees of unsaturation. Two compounds were identified as olefins (δ_C_ 153.0 (C); 127.0 (CH) (δ_H_ 5.88 brt, *J* = 2.5); 150.1 (C); 111.4 (CH_2_) (δ_H_ 4.70 s, 4.76 s)) and three others were assigned to a trisubstituted epoxide (δ_C_ 61.3 (CH) (δ_H_ 3.06 t, *J* = 6.5); δ_C_ 63.7 (C)) and a bicyclic structure. IR absorption at 3310 cm^−1^ and at 1714 and 1040 cm^−1^ suggested the presence of hydroxy and exomethylene groups, respectively.

**Figure 2:**
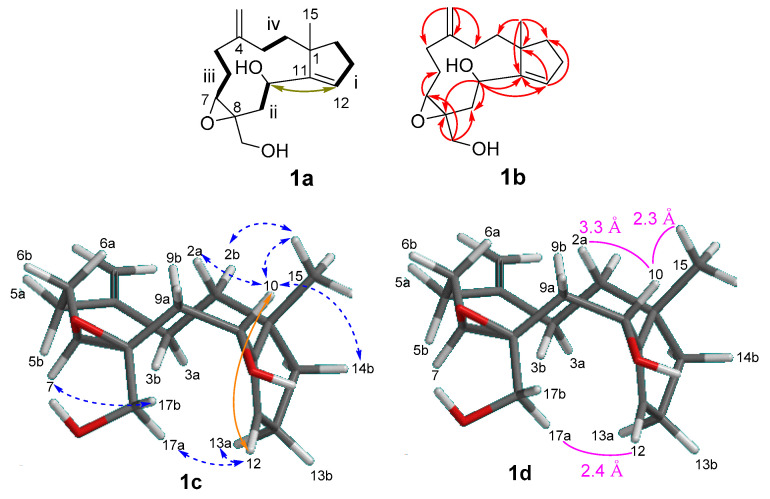

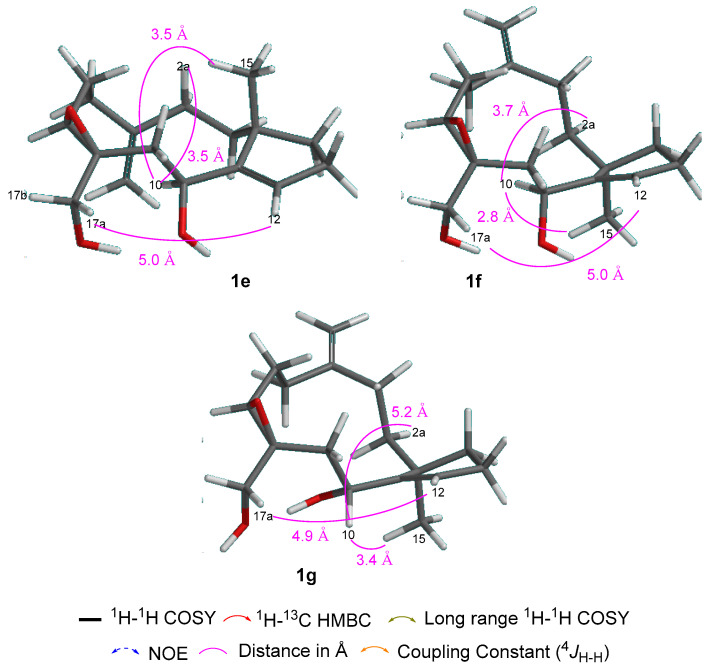
Key: COSY (**1a**), HMBC (**1b**), NOE (**1c**), correlations and a long-range coupling constant (^4^*J*_H-H_), as well as the distance between atoms (**1d**), with **a** computer-generated model of **1** (energy minimized: 1S*, 7S*, 8S*, 10R* for (**1c**); 1S*, 7S*, 8S*, 10S* for (**1e**); 1R*, 7S*, 8S*, 10S* for (**1f**); 1R*, 7S*, 8S*, 10R* for (**1g**), obtained from calculations with molecular mechanics MMFF).

Four spin systems **i**–**iv** (**1a**; Figure 2) were disclosed by inspecting ^1^H–^1^H COSY cross peaks: (**i**) a trisubstituted double bond next to two methylenes (δ_H_ 5.88, 2.31, 2.10, 1.84, 1.63; H-12 to H-14), (**ii**) an oxymethine connected to a methylene (δ_H_ 2.20, 4.40; H-9 to H-10), (**iii**) the epoxide methine next to two methylenes (δ_H_ 2.42, 2.22, 1.73, 3.06; H-5 to H-7), and (**iv**) two methylenes (δ_H_ 1.63, 1.50, 2.10, 1.73; H-2 to H-3). A small coupling (^4^*J*_H-H_ 1.3 Hz) between H-10 and H-12 with a COSY cross peak supported the presence of an allylic alcohol in Figure 2 (**1a**). HMBC correlations for H-10/C-8, 9, 11, 12 and H-12/C-1, 10, 11, 13, 14 confirmed the connection of spin systems **i** and **ii**. The trisubstituted epoxide was placed at C-7 and C-8, connecting spin systems **ii**, **iii,** and a primary alcohol (δ_H_ 3.38, 4.07) by observing HMBC correlations for H-7/C-6, 17; H-10/C-8; H-9/C-7, 8, 17; H-17a,b/C-7, 8, 9. Spin systems **iii** and **iv** were connected through an exomethylene (δ_H_ 4.70 s, 4.76 s; δ_C_ 153.0, 111.4) placed at C-4, as HMBC correlations H-16a,b/C-3, 4, 5 were observed. Finally, correlations from H_3_-15 to C-1, 2, 11, 14 supported the connection of spin systems **i** and **iv,** confirming the planar structure of **1** as a trinor-dolabellane diterpenoid.

The relative stereochemistry of **1** was tentatively assigned as follows, based on positive NOEs (**1c**; Figure 2). By observing a strong NOE between H-7 and H-17b (**1c**; Figure 2), chirality at the epoxide was revealed to be 7S*, 8S*, as in similar structural units [6,11]. Therefore, four possible stereoisomers—**1c** (1S*, 7S*, 8S*, 10R*), **1e** (1S*, 7S*, 8S*, 10S*), **1f** (1R*, 7S*, 8S*, 10S*), and **1g** (1R*, 7S*, 8S*, 10R*)—were further considered. Figure 2 shows the energy-minimized conformations of **1c** and **1e**–**1g** after molecular mechanics (MMFF) calculation. Of four possibilities, **1c** was the most likely structure because a positive NOE was observed between H-17a and H-12 within a reasonable distance (Figure 2), while other candidates were expected to have longer distances (~5 Å). The angular methyl H-15 at δ_H_ 1.09 partially supported this conformation, showing NOEs for H-15/H-2b, 10 (Figure 2). The more downfield-shifted signal for the C-15 (δ 1.09) of **1** compared to that (δ 0.85) of the stolonidiol of **4** [6,11] may be due to the absence of the epoxide ring and the presence of an olefin at the ring junction.

**Figure 3:**
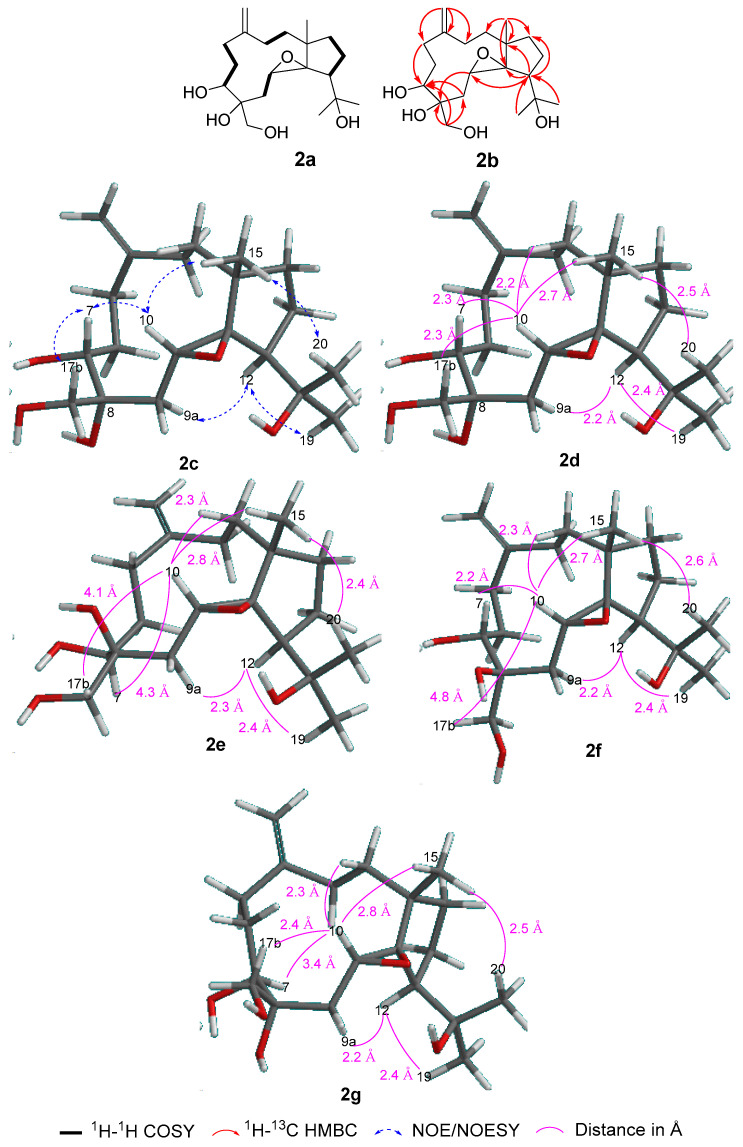
Key: COSY (**2a**), HMBC (**2b**), NOE (**2c**), correlations and distance between atoms (**2d**) with **a** computer-generated model of **2** (energy-minimized 1*S*, 7*R*, 8*R*, 10*R*, 11*R*, 12*S* for (**2c**); 1*S*, 7*S*, 8*S*, 10*R*, 11*R*, 12*S* for (**2e**); 1*S*, 7*R*, 8*S*, 10*R*, 11*R*, 12*S* for (**2f**); 1*S*, 7*S*, 8*R*, 10*R*, 11*R*, 12*S* for (**2g**), obtained from calculations with MMFF).

**Figure 4:**
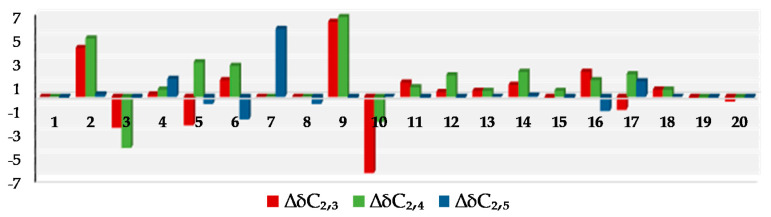
Comparative analysis of ^13^C chemical shifts between **2** and **3**, **4, 5**.

Using HRESIMS and NMR, it was determined that sangiangol B (**2**), [α]_D_^27^ + 15, has the molecular formula C_20_H_34_O_5_, with an additional oxygen and two hydrogen atoms compared to stolonidiol (**4**). Furthermore, four degrees of unsaturation in **2** indicated a similarity to clavinflol B (**5**) [6,12], a chlorohydrin analog. A detailed 2D NMR (Figure 3) analysis of **2** revealed that the major differences between **2**, **4,** and **5** were the chemical shifts at C-7 (*δ*_H_ 3.56, d (11.4); *δ*_C_ 72.9 for **2** and *δ*_H_ 3.96, d (11.5); *δ*_C_ 67.2 for **5**) [6,12] and at C-8 (*δ*_C_ 75.3 for **2** and 63.2 for **4**) [6,11] (Figure 4). With the NMR chemical shifts and high-resolution mass spectrometry (HRMS) data, compound **2** contained 1,2-diol at C-7 and C-8 for an epoxide in stolonidiol (**4**) or for a chlorohydrin in clavinflol B (**5**) [6,12]. Key HMBC correlations for H-7/C-8, 17; H-17/C-7, 8; H-9/C-7; H-10/C-8 further confirmed the position of the diol, establishing the planar structure.

Of the six stereocenters of sangiangol B (**2**), three can be confirmed as 1*S*, 11*R*, and 12*S* by comparing the ^13^C-NMR data for the cyclopentane moiety (C-1, C-11~14) with those of **3**, **4**, and **5** (Figure 4) [6,11,12] and by observing the rotation value [α]_D_-37.9 of co-isolated stolonidiol (**4**) and the value [α]_D_-31 of that reported in **4**, the absolute stereochemistry of which was established by X-ray crystallography [11]. Chirality at C-10 was shown to have the same *R* configuration as stolonidiol (**4**), based on a positive NOE between H-10 and H-15. Among the four possible structures—**2d**: 7*R*,8*R*, **2e**: 7*S*,8*S*, **2f**: 7*R*,8*S*, and **2g**: 7*S*,8*R*—the distances for H-10/H-7 and H-10/H-17b with energy-minimized conformations were compared, as in Figure 3. Both **2e** and **2f** were eliminated due to the relatively long distances of H-10/H-17b. However, as two candidates, **2d** and **2g,** accorded with the spectral data, the configuration at C-8 was then revealed, while only C-7 remained to be solved.

**Figure 5:**
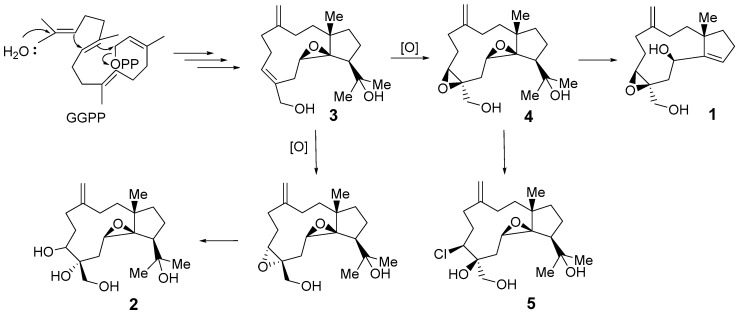
A possible biosynthetic pathway for sangiangols A (**1**) and B (**2**).

Furthermore, the biosynthesis of stolonidiol-related molecules can be proposed, as in Figure 5. Geranylgeranyl pyrophosphate (GGPP) is a well-known starting material for diterpenoids [13]. Sangiangol A (**1**) could be derived from stolonidiol (**4**) through a series of degradation and epoxide ring-opening reactions, while sangiangol B (**2**) could be derived from sangiangol C (**3**) through an epoxidation reaction. Moreover, sangiangol C (**3**) could be the precursor of stolonidiol (**4**). Unfortunately, attempts to prepare α-methoxy-α-trifluoromethylphenylacetic acid (MTPA) esters for the determination of the absolute configurations of both molecules failed because of their instability and the small quantities of these compounds available.

All isolated compounds (**1**–**8**) were evaluated for cytotoxicity against NBT-T2 rat bladder epithelial cells (Table 2). New entities **1** and **2** showed weak toxicity at 5 and 10 μg/mL, respectively, while known molecules **3**–**8** showed moderate and weak toxicity at 10, 1, 0.5, 10, 1, and 10 μg/mL, respectively. From the structure–activity relationship of stolonidiol derivatives, two epoxide rings or a chlorine atom are required for their cytotoxicity, as in **4** (stolonidiol) and **5** (clavinflol B).

## 3. Materials and Methods

### 3.1. General Methods

The optical rotations were obtained with a JASCO P-1010 digital polarimeter. The ^1^H and ^13^C-NMR spectra were recorded on a JEOL α 500 FT NMR spectrometer. The chemical shifts were expressed in *δ* (ppm) and the coupling constants (*J*) in Hz. The electrospray ionization mass spectrometry (ESIMS) data were obtained on a PE QSTAR mass spectrometer and the infrared (IR) spectra were recorded on a DR 8020 Shimadzu spectrophotometer. The HPLC was performed on a Hitachi L-6000 pump equipped with a Shodex RI-101 monitor and a Hitachi L-4000 UV detector, using a Cosmosil 5C_18_AR-II (5 µm) or a Mightysil RP-18 (5 µm) column. Merck silica gel 60 (0.063–0.20 mm) was used for column chromatography. The analytical thin layer chromatography (TLC) was performed on commercial silica gel 60 F_254_ visualized with vanillin–EtOH-1% H_2_SO_4_. All solvents used were reagent grade.

### 3.2. Animal Material

A marine soft coral (AA-C31) was collected from Krakatau Island, Banten, Indonesia at 10–15 m depth by hand, while scuba diving. It was then stored in EtOH. The specimen was identified as *Anthelia* sp. by one of us (J.T.).

### 3.3. Extraction and Isolation

The fresh soft coral specimen (wet weight, 121 g) stored in EtOH was extracted four times using Me_2_CO (4 × 150 mL). The four solutions were pooled and concentrated under vacuum, and the resulting residue was partitioned between EtOAc and H_2_O to obtain a cytotoxic lipophilic extract (1.57 g, NBT-T2 1 μg/mL). The whole extract was separated on a Si gel 60 column by eluting stepwise with hexane–EtOAc–MeOH to afford 19 fractions. The second fraction (9.7 mg) was further separated using normal phase silica HPLC (hexane 100%) to give cembrane A (**6**) [13] (2.3 mg). The eighth fraction was purified on reversed phase HPLC to give sangiangol C (**3**) [14] (1.0 mg). The ninth fraction was repeatedly separated by HPLC (first, reversed-phase C_18_ (RP_18_), MeOH–H_2_O, 5:2; second, Si 60, CH_2_Cl_2_–EtOAc, 7:3) to afford kericembrenolide E (**7**) [15] (2.7 mg), stolonidiol (**4**) [6,11] (3.5 mg), and clavinflol B (**5**) [6,12] (1.3 mg). The tenth fraction was subfractionated on RP_18_ HPLC to give cembrenolide (**8**) [16] (1.7 mg), sangiangol A (**1**) (1.3 mg), and stolonidiol (**4**) (103.7 mg). Finally, the most polar fraction was repeatedly separated on reversed-phase HPLC (first, RP_18_: MeOH–H_2_O, 2:1 second, RP_18_, MeOH–H_2_O, 4:5) to give sangiangol B (**2**) (1.4 mg).

#### 3.3.1. Sangiangol A (**1**)

Colorless oil; [α]_D_^27^ + 20 (*c* 0.09, CHCl_3_); IR (KBr) ν_max_ 3418, 2965, 1683, 1645, 1456, 1378, 1168, 1064 cm^−1^; ^1^H and ^13^C-NMR (see Table 1 and Table 2); HRESIMS *m*/*z* 301.1658 [M + Na]^+^ (calculated (calcd) for C_17_H_26_O_3_Na 301.1779).

#### 3.3.2. Sangiangol B (**2**)

Colorless oil; [α]_D_^27^ + 15 (*c* 0.14, CHCl_3_); IR (KBr) ν_max_ 3418, 2965, 1645, 1456, 1378, 1168, 1064 cm^−1^; ^1^H and ^13^C-NMR (see Table 1 and Table 2); HRESIMS *m*/*z* 377.2230 [M + Na]^+^ (calcd for C_20_H_34_O_5_Na 377.2304).

### 3.4. Cytotoxicity Assay

NBT-T2 rat bladder epithelial cells (BRC-1370) purchased from RIKEN (Tsukuba, Ibaraki, Japan) were cultured under a standard protocol using Dulbecco’s modified Eagle’s medium (DMEM). The cells were seeded in 1 mL of modified Eagle’s media supplemented with 10% heat-inactivated fetal bovine serum, streptomycin, amphotericin B, and glutamic acid. The cells were exposed to graded concentrations of the new and known compounds, as well as their fractions at 37 °C, for 72 h and observed under a microscope to observe the effects at 48 and 72 h.

## Figures and Tables

**Table 1:** ^1^H NMR data for compounds **1** and **2** in CDCl_3_.

Position	Sangiangol A (1)				Sangiangol B (2)			
	δ_C_ *	mult.	δ_H_ **	*J* in Hz	δ_C_ *	mult.	δ_H_ **	*J* in Hz
1	50.2	C			44.7	C		
2a	38.4	CH_2_	1.63	m ***	42.8	CH_2_	1.97	m
2b			1.50	ddd (14.4, 11.6, 7.2)			1.25	m ***
3a	29.6	CH_2_	2.10	m ***	25.0	CH_2_	2.09	dt (15.4, 10.0)
3b			1.73	m ***			1.64	dd (13.3, 10.0)
4	150.1	C			149.3	C		
5a	30.8	CH_2_	2.42	dt (14.6, 5.4)	34.3	CH_2_	2.45	td (13.6, 4.6)
5b			2.22	dd (14.6, 7.7)			2.29	brdd (13.6, 4.6)
6a	24.4	CH_2_	1.73	m ***	27.4	CH_2_	1.79	m
6b			1.73	m ***			1.50	m
7	61.3	CH	3.06	t (6.5)	72.9	CH	3.56	d (11.4)
8	63.7	C			75.3	C		
9a	36.0	CH_2_	2.20	dd (15.7, 3.0)	33.6	CH_2_	1.95	dd (4.5, 2.7)
9b			2.00	dd (15.7, 6.3)			1.92	dd (5.5, 1.6)
10	65.1	CH	4.40	dd (6.3, 1.3)	54.5	CH	3.02	dd (5.4, 2.7)
11	153.0	C			76.7	C		
12	127.0	CH	5.88	brt (2.5)	50.2	CH	2.18	d (10.8, 2.0)
13a	29.7	CH_2_	2.31	m ***	27.8	CH_2_	1.89	m
13b			2.10	m ***			1.60	m
14a	36.7	CH_2_	1.84	ddd (12.7, 9.0, 7.1)	39.0	CH_2_	1.79	m
14b			1.63	m ***			1.74	m
15	27.4	CH_3_	1.09	s	24.0	CH_3_	0.84	s
16a	111.4	CH_2_	4.76	s	112.7	CH_2_	4.96	s
16b			4.70	s			4.79	s
17a	67.2	CH_2_	4.07	d (12.2)	67.2	CH_2_	3.89	d (11.4)
17b			3.38	d (12.2)			3.52	d (11.4)
18					75.2	C		
19					29.6	CH_3_	1.21	s
20					26.0	CH_3_	1.26	s

* Assigned by DEPT and 2D NMR (HSQC and HMBC) experiments. ** Assigned by 2D NMR (COSY, HSQC, and HMBC) experiments. *** Overlapping signals.

**Table 2:** Cytotoxicity of compounds **1**–**8** against NBT-T2 rat bladder epithelial cells.

Compound	Concentration (µg/mL)
1	5
2	10
3	10
4	1
5	0.5
6	10
7	1
8	10
